# Anti-Inflammatory and Antioxidant Properties of β-Sitosterol in Copper Sulfate-Induced Inflammation in Zebrafish (*Danio rerio*)

**DOI:** 10.3390/antiox12020391

**Published:** 2023-02-06

**Authors:** Peng Zhang, Naicheng Liu, Mingyang Xue, Mengjie Zhang, Wei Liu, Chen Xu, Yuding Fan, Yan Meng, Qinghua Zhang, Yong Zhou

**Affiliations:** 1 Key Laboratory of Exploration and Utilization of Aquatic Genetic Resources, Ministry of Education, Shanghai Ocean University, Shanghai 201306, China; 2 Yangtze River Fisheries Research Institute, Chinese Academy of Fishery Sciences, Wuhan 430223, China; 3 National Pathogen Collection Center for Aquatic Animals, Shanghai Ocean University, Shanghai 201306, China

**Keywords:** β-sitosterol, zebrafish (*Danio rerio*), oxidative stress, anti-inflammation, antioxidant

## Abstract

β-Sitosterol, which is used extensively in pharmaceuticals, nutraceuticals, and cosmetics, has high nutritional value along with immunomodulatory and anti-inflammatory properties. In this study, we investigated the antioxidant and anti-inflammatory effects of β-sitosterol in zebrafish and explored the associated molecular mechanisms. In an in vivo antioxidant experiment, zebrafish (*Danio rerio*) larvae were treated with different concentrations of β-sitosterol and then exposed to a nonlethal concentration of CuSO_4_ to induce oxidative stress. Treatment with β-sitosterol at 70 or 100 μg/mL significantly reduced CuSO_4_-induced oxidative stress in the zebrafish, demonstrating the strong antioxidant activity of β-sitosterol. Treatment with β-sitosterol protected zebrafish larvae against oxidative damage from CuSO_4_ by upregulating the expressions of *sod* and *gpx4b*. In a zebrafish model of inflammation, pretreatment with β-sitosterol before CuSO4 exposure inhibited neutrophil recruitment and damage to lateral line neuromasts, indicating a potent anti-inflammatory effect derived from reductions in the expressions of *il-8* and *myd88*. The results demonstrate the antioxidative and anti-inflammatory activities of β-sitosterol and suggest that β-sitosterol may be useful for the treatment of various inflammatory diseases.

## 1. Introduction

Inflammation is the root cause of many chronic diseases, such as obesity, hypertension, atherosclerosis, cancer, and Alzheimer’s disease [1]. In the presence of inflammatory signals, neutrophils first infiltrate into the site of infection, injury, or inflammation. Neutrophils are the major source of reactive oxygen species (ROS) during inflammation [2]. Therefore, oxidative stress develops and further exacerbates inflammation [3]. Conversely, when oxidative stress occurs, the overproduction of ROS and nitrogen species (NOS) will drive the inflammatory response by inducing the expression of proinflammatory genes [4]. Thus, the inflammatory process can exacerbate oxidative stress and vice versa. Traditional anti-inflammatory agents can cause severe side effects, such as hemorrhagic gastritis, upper gastrointestinal hemorrhage, gastrointestinal toxicity (diarrhea/colitis), and hypertension [5,6], limiting the use of these drugs [7]. While oxidative stress and inflammation are interdependent, the treatment of primary abnormalities alone is not always successful [3]. Thus, it is crucial to find effective anti-inflammatory drugs that simultaneously inhibit ROS production and mediate inflammation; this is considered a promising strategy for preventing and treating diseases associated with chronic inflammation. Oxidative and inflammatory responses can be easily induced and visualized in zebrafish, especially those in the early developmental stages [8]. Copper is an essential micronutrient that can also be toxic to many types of cells at certain levels [9]. Excessive inorganic copper in the environment can disrupt the copper balance in zebrafish [10], leading to elevated serum copper levels and triggering an oxidative stress responses. This ultimately results in an oxidative stress damage-mediated inflammatory response [11]. In addition, the zebrafish *(Danio rerio*) fish embryo toxicity (FET) assay is a commonly used methodology for determining the toxicity of environmental pollutants [12].

Phytosterol is a natural active substance that has been hailed as “the key of life” [13]. The most common and abundant phytosterols, including β-sitosterol, exhibit important physiological functions [14]. In animal models, β-sitosterol was found to alleviate dextran sulfate sodium-induced colitis in mice; β-sitosterol not only enhanced the body weight and improved the colonic morphology in the mice, it also decreased the mRNA expressions of proinflammatory cytokines in the colon in a dose-dependent manner [15]. In vivo animal studies showed that β-sitosterol has strong antigenotoxic effects, while in vitro tests revealed a dose-dependent antioxidant effect of β-sitosterol [16]. However, few studies have evaluated the effects of β-sitosterol in fish, and little is known about the mechanism of action of β-sitosterol in fish. In this study, we used CuSO_4_ to induce an inflammatory response through an oxidative stress reaction in zebrafish and investigated the antioxidant and anti-inflammatory effects of β-sitosterol. We also investigated the molecular mechanisms of β-sitosterol. This study provides a scientific basis for the application of β-sitosterol in traditional medicine and aquatic feed additives.

## 2. Materials and Methods

### 2.1. Fish and Experimental Conditions

The wild-type AB zebrafish (*Danio rerio*) and transgenic zebrafish line *Tg* (*lyz*: *DsRED2*) were purchased from China Zebrafish Resource Center (CZRC, Wuhan, China). *Tg* (*lyz*: *DsRed2*) was used for the neutrophil imaging experiments. The zebrafish were raised and maintained according to the guidelines available at http://zfin.org/ (accessed on 1 June 2022). The zebrafish were fed three times each day. The day before mating, males and females were placed in breeding tanks. Mating and spawning occurred within 1 h after turning on the lights in the morning. The embryos were picked under a microscope, and the selected fertilized eggs were placed in E3 medium at 28 °C. The media were replaced after 24 h, and all experiments were carried out using zebrafish larvae at 3 days postfertilization (dpf). All animal experiments were approved by the Animal Experimental Ethical Inspection of Laboratory Animal Centre, Yangtze River Fisheries Research Institute, Chinese Academy of Fishery Sciences (ID number: YFI 2022-zhouyong-07).

### 2.2. Main Reagents

β-Sitosterol was purchased from Shanghai Yuanye Bio-Technology Co., Ltd. (Shanghai, China). 2,2-Diphenyl-1-picrylhydrazyl (DPPH), quercetin, 2′,7′-dichlorodihydrofluorescein diacetate (H_2_DCFDA), pyrocatechol violet (PV), copper sulfate pentahydrate (CuSO_4_·5H_2_O), and methyl cellulose were purchased from Merck Co., Ltd. (Shanghai, China). Dimethylformamide (DMSO) was purchased from Sangon Biotech Co., Ltd. (Shanghai, China). The E3 medium was purchased from Nanjing EzeRinka Biotechnology Co., Ltd. (Nanjing, China). The components of E3 were NaCl (29.4 g/100 mL), KCl (1.27 g/100 mL), CaCl_2_·2H_2_O (4.85 g/100 mL), and MgSO_4_·7H_2_O (8.13 g/100 mL) [17].

### 2.3. Experimental Design

#### 2.3.1. Preparation of Test Samples

Dimethyl sulfoxide (DMSO) was used as a solvent. β-Sitosterol stock solution (200 µg/mL) was prepared in DMSO. Working solutions were prepared by diluting the stock solution with E3 medium. The experimental concentrations of β-sitosterol were 0, 5, 20, 50, 70, and 100 µg/mL. The stock solution of H_2_DCFDA (20 µg/mL) was prepared in DMSO and frozen at −20 °C. The working solutions were prepared on the day of the experiment.

#### 2.3.2. Chelating Ability of β-Sitosterol for Cu^2+^

The chelating ability of β-sitosterol for Cu^2+^ was evaluated according to the method described by Santos et al. [18] using PV as the chromogen agent. A 30 µL solution of β-sitosterol at different concentrations (5, 20, 50, 75, and 100 µg/mL) or water (control) were mixed with 200 µL of sodium acetate buffer (50 mmol/L). Then, 30 μL of 100 mg/L CuSO_4_ ·5H_2_O solution was added to each group for 2 min. After 2 min, 2 mmol/L 8.5 μL catechol violet solution was added to initiate the reaction. All reaction mixtures were shaken at 25 °C for 10 min, and then the absorbance was measured at 632 nm using an UV-Vis spectrophotometer (Palo Alto, CA, USA). The Cu^2+^ chelating ability of β-sitosterol was calculated as follows: Cu^2+^ chelating ability of β-sitosterol (%) = (Abs control − Abs sample) × 100/Abs control.

#### 2.3.3. Antioxidant Effect of β-Sitosterol In Vitro

The antioxidant activity was measured in vitro based on the DPPH radical scavenging, following Cheng-zhong et al. [19]. A 20 µL solution of β-sitosterol at different concentrations (5, 20, 50, 75, and 100 µg/mL) was added to a 180 µL methanol solution of 0.1 mM DPPH. Methanol was used as a blank, and quercetin was used as a positive control. The reaction solution was shaken at room temperature for 30 min in dark. Then the absorbance was measured at 517 nm using a UV-Vis spectrophotometer. The percent inhibition of DPPH radical scavenging capacity was calculated as follows: scavenging activity (%) = (Abs control − Abs sample) × 100/(Abs control).

#### 2.3.4. In Vivo Antioxidant Test

The production of ROS was detected by a H_2_DCFDA assay [20]. Zebrafish embryos (3 dpf) were transferred to a six-well plate (10 larvae per well) containing 940 μL E3 medium. The zebrafish larvae were treated with β-sitosterol (0, 5, 20, 50, 70, or 100 μg/mL) or 100 μM quercetin (positive control) for 1 h. The larvae were then treated with 10 μM CuSO_4_ for 20 min to induce oxidative stress [20], rinsed with fresh E3 medium, incubated with H_2_DCFDA for 1 h in the dark at 28 °C, and again rinsed in fresh E3 medium. The zebrafish larvae were anesthetized with 2% MS-222, immobilized in 3% methylcellulose, and observed under a fluorescence microscope (Olympus, Tokyo, Japan) and photographed. The average fluorescence intensity was quantified using Image J 1.48 software. The larvae treated with CuSO_4_ alone served as the negative control, while the larvae treated with quercetin were the positive control.

#### 2.3.5. Neutrophil Migration Assay

The neutrophil migration assay was performed according to Reference [21] using the *Tg* (*lyz*: *DsRED2*) zebrafish larvae (3 dpf) as the study subjects. The zebrafish larvae were treated with β-sitosterol (0, 5, 20, 50, 70, or 100 μg/mL) for 1 h followed by 20 µM CuSO_4_ for 1 h [20], with 10 larvae in each group. The presence of neutrophils was monitored at the lateral line to evaluate the degree of inflammation. The neutrophil migration was quantified by calculating the number of labeled cells detected in the lateral line region. The larvae treated with DMSO alone served as the blank control, while the larvae treated with CuSO_4_ alone were the negative control.

#### 2.3.6. Fluorescence Assay Quantification Method

For the in vivo antioxidant test, zebrafish fluorescence images were collected using the same magnification and exposure time. The individual zebrafish larvae fluorescence intensity was quantified using the Image J program. For the neutrophil migration assay, the number of neutrophils in the neuromast was quantified using the Image J program.

#### 2.3.7. Quantitative Real-Time Polymerase Chain Reaction (RT-qPCR)

The transcriptome and RT-qPCR analysis methods referred to Reference [20]. The zebrafish larvae (3 dpf) were transplanted into a six-well plate containing 940 μL E3 medium (30 larvae per well). The 30 hatched larvae in each group were treated with different doses of β-sitosterol (0, 5, 20, 50, 70, or 100 μg/mL) for 1 h and then exposed to 10 μM CuSO_4_ (a concentration that induces oxidative stress and inflammation [11]) for 24 h. The larvae were collected after 24 h and stored at −80 °C for RNA extraction. Total RNA was extracted from the larvae using TRIzol reagent (Invitrogen, Carlsbad, CA, USA) [22]. The concentration (A260) and purity (A260/A280 and A260/A230) of the RNAs were measured using a NanoDrop 1000 instrument (Thermo Fisher Scientific). The RNA integrity was confirmed by electrophoresis in a 1.5% agarose denaturing gel and reverse-transcribed into cDNA using a cDNA reverse transcription kit (Trans Gen Biotech, Wuhan, China). The cycling conditions for RT-qPCR were 95 °C for 10 min followed by 40 cycles of 95 °C for 30 s and 60 °C for 30 s. The results were obtained using the 2^−ΔΔct^ method [23]. The specific primers used are listed in Table 1.

#### 2.3.8. Data Presentation and Statistical Analyses

The normally distributed data are expressed as the mean ± standard deviation. The differences between treatments were determined by *t*-test or one-way analysis of variance (ANOVA), as well as the least significant difference test. A probability level of 5% (*p* < 0.05) was considered significant. When the normality test failed, the Kruskal–Wallis nonparametric one-way ANOVA was used, and the differences between groups were determined by the Mann–Whitney test. The nonparametric data are presented using Tukey’s boxplot, where the bottom and top of the box are the minimum and maximum values, respectively.

## 3. Results

### 3.1. Chelating Ability of β-Sitosterol for Cu^2+^

**Figure 1:**
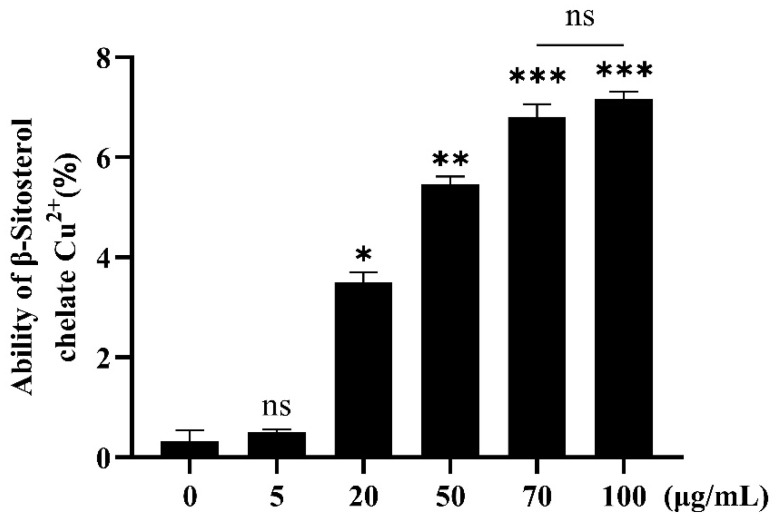
Metal chelating ability of β-sitosterol for Cu^2+^. Each bar represents the mean ± standard error for three different experiments performed in triplicate. ns, no statistical significance, * *p* < 0.05, ** *p* < 0.01, and *** *p* < 0.001 compared with the control group (0 μg/mL β-sitosterol).

In this experiment, CuSO_4_ solution was used as the Cu+ source. The chelating ability of β-sitosterol for Cu^2+^ increased with an increasing β-sitosterol concentration (Figure 1). The ability of β-sitosterol to complex with Cu^2+^ increased with an increasing β-sitosterol concentration. In terms of the chelation ability, the groups treated with 5 and 0 μg/mL β-sitosterol did not show a significant difference, while the remaining experimental groups were significantly different from the 0 μg/mL group. At the highest concentration of β-sitosterol (100 μg/mL), the chelating ability of β-sitosterol for Cu^2+^ was only 7%. No significant difference in the chelation ability was found between the groups treated with β-sitosterol at 70 and 100 μg/mL (Figure 1). Thus, β-sitosterol had a weak chelating activity for Cu^2+^, indicating that metal chelation was not important in the subsequent experiments.

### 3.2. Antioxidant Effect of β-Sitosterol

#### 3.2.1. In Vitro Antioxidant Effect of β-Sitosterol

**Figure 2:**
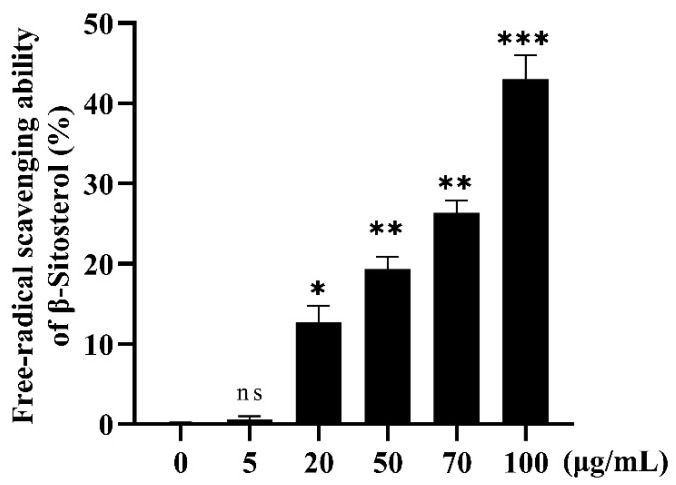
Free radical scavenging rates of β-sitosterol at different concentrations. Each bar represents the mean ± standard error for three different experiments performed in triplicate. ns, no statistical significance, * *p* < 0.05, ** *p* < 0.01, and *** *p* < 0.001 compared with the control group (0 µg/mL β-sitosterol).

The free radical scavenging ability of β-sitosterol was determined by the DPPH assay. The scavenging rate of the free radicals increased with an increasing β-sitosterol concentration in the experimental concentration range (Figure 2). With the exception of the group treated with 5 μg/mL β-sitosterol, the β-sitosterol-treated groups showed a significantly different free radical scavenging ability compared with the control group (0 μg/mL β-sitosterol). A the β-sitosterol concentration of 100 μg/mL, the scavenging rate was 43%, indicating that β-sitosterol had a strong ability to scavenge DPPH (Figure 2).

#### 3.2.2. In Vivo Antioxidant Effect of β-Sitosterol

**Figure 3:**
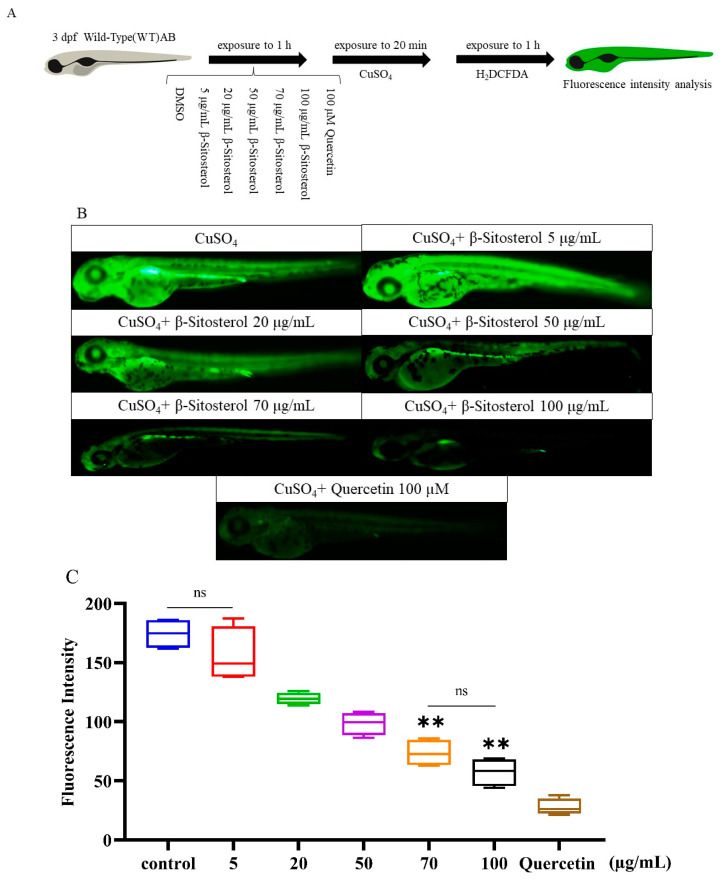
Inhibitory effect of β-sitosterol on the CuSO_4_-induced production of ROS in zebrafish larvae: (**A**) flowchart of the experiments; (**B**) representative fluorescence images of zebrafish in different experimental groups; (**C**) fluorescence intensity in juvenile zebrafish in different experimental groups compared with the group treated with CuSO_4_ alone. ns, no statistical significance, ** *p* < 0.01 compared with the control group (*n* = 4).

The ROS produced in zebrafish were detected using the fluorescent probe H_2_DCFDA, and quercetin was used as a positive control. Quercetin (100 μM) effectively inhibited the formation of ROS (Figure 3B), and the fluorescence intensity in the positive control was significantly reduced compared to that in the CuSO_4_ group (Figure 3C). Similar to quercetin, β-sitosterol at concentrations of 50, 70, and 100 μg/mL significantly reduced the ROS fluorescence intensity compared to the control. Treatment with β-sitosterol at 5 μg/mL did not have a significant effect on the fluorescence intensity compared to the control group (Figure 3C), indicating that this low concentration of β-sitosterol did not obviously inhibit ROS production.

#### 3.2.3. Effect of β-Sitosterol on the Expressions of the Antioxidant Genes *sod* and *gpx4b*

**Figure 4:**
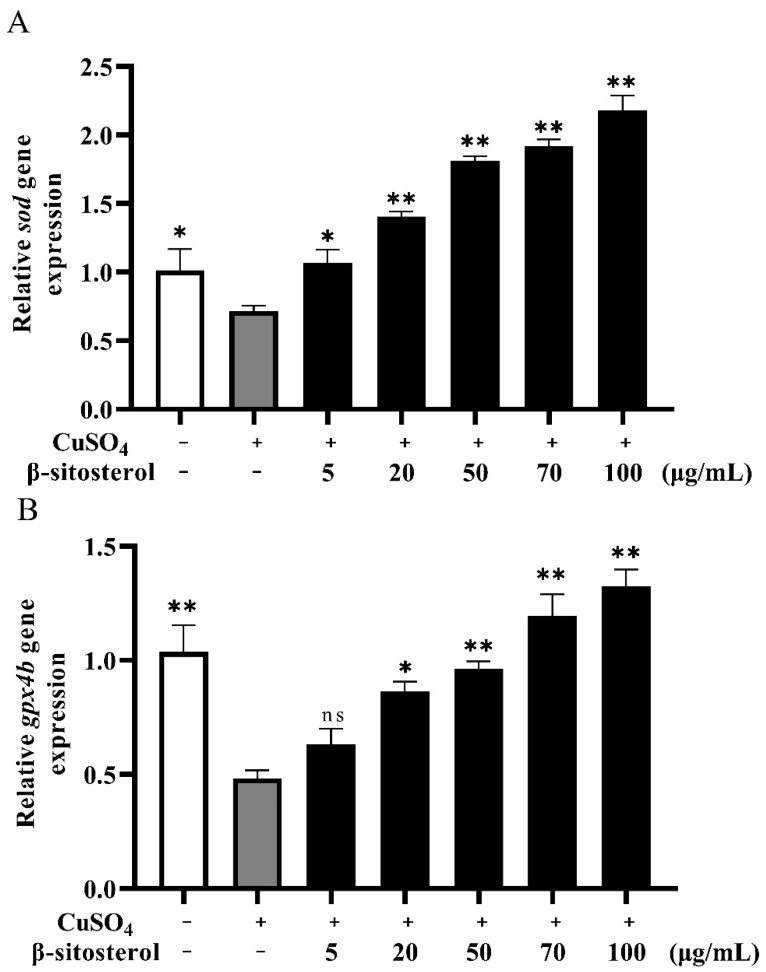
Relative expressions of antioxidant-related genes in zebrafish larvae treated with CuSO_4_ and β-sitosterol for 24 h: (**A**) relative expression of the *sod* gene; (**B**) relative expression of the *gpx4b* gene. These data are expressed as the mean ± standard error based on three biological replicates. ns, no statistical significance, * *p* < 0.05 and ** *p* < 0.01 compared with the group treated with CuSO_4_ alone.

We used RT-qPCR to analyze the mRNA expression levels of two antioxidant genes, *sod* and *gpx4b*, in the different treatment groups. Compared with the control group, the expressions of *sod* and *gpx4b* were significantly reduced in the presence of CuSO_4_ (Figure 4). However, after 24 h of β-sitosterol treatment, the expressions of *sod* and *gpx4b* were significantly upregulated compared with the CuSO_4_ group, with significant upregulation observed in the 50, 70, and 100 μg/mL β-sitosterol treatment groups for *sod* (Figure 4A) and in the 70 and 100 μg/mL β-sitosterol treatment groups for *gpx4b* (Figure 4B).

### 3.3. Anti-Inflammatory Effect of β-Sitosterol in Zebrafish

#### 3.3.1. Effect of β-Sitosterol on Neutrophil Migration

**Figure 5:**
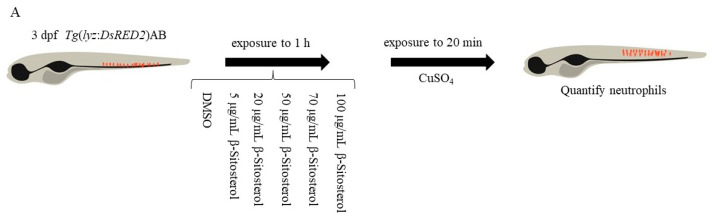

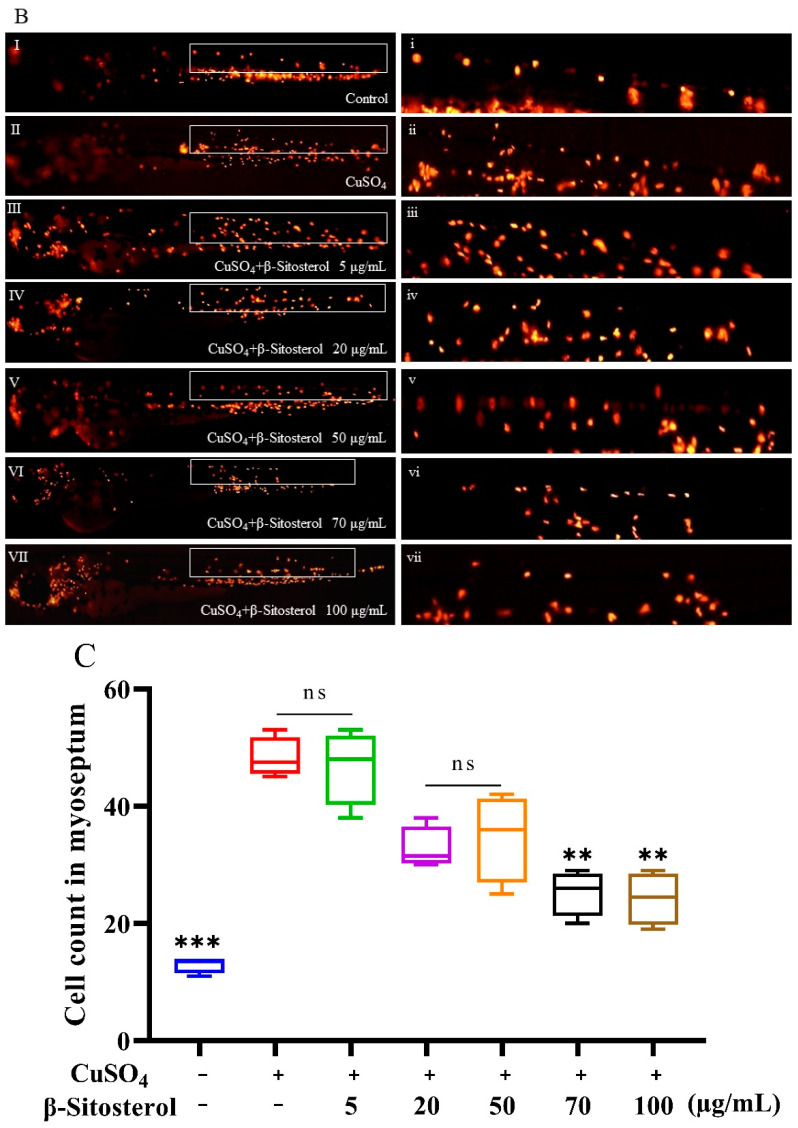
Effect of β-sitosterol on CuSO_4_-induced inflammatory response in zebrafish: (**A**) flowchart of the experiments; (**B**) neutrophil migration in zebrafish after treatment; (**C**) quantification of the number of neutrophils aggregated at the lateral line after CuSO_4_ treatment. Data are expressed as the mean ± standard deviation. ns, no statistical significance, ** *p* < 0.01, and *** *p* < 0.001 compared with the group treated with CuSO_4_ alone (*n* = 4).

The effect of β-sitosterol on the CuSO_4_-induced inflammatory response was evaluated using the transgenic *Tg* (*lyz*: *DsRED2*) zebrafish model. In the control group, most neutrophils were located in the tail hematopoietic tissues of the ventral stem and tail (Figure 5B). In the group treated with CuSO_4_ alone, the neutrophils migrated to the horizontal midline, as indicated by the white square box in Figure 5B(i), and formed clusters near the lateral line nerve hypertrophy. As shown in Figure 5B(i–vii), which respectively correspond to Figure 5B(I–VII), as the concentration of β-sitosterol increased, the number of neutrophils that migrated to the lateral line after the CuSO_4_ treatment gradually decreased. The neutrophils in the white boxes in Figure 5B(i–vii) were quantified using Image J software. The number of neutrophils was significantly different between the group treated with CuSO_4_ alone and the control group (Figure 5C). However, there was no significant difference with the group treated with 5 μg/mL β-sitosterol (Figure 5C). No significant difference in neutrophil migration was found between the groups treated with 20 and 50 μg/mL β-sitosterol (Figure 5C). Compared with the group treated with CuSO_4_ alone, the groups pretreated with β-sitosterol at 70 and 100 μg/mL showed significant differences in neutrophil migration (Figure 5C). Thus, the effect on neutrophil migration became stronger as the concentrations of β-sitosterol increased, suggesting that β-sitosterol significantly inhibited neutrophil migration to the lateral line.

#### 3.3.2. Effects of β-Sitosterol on the Expressions of *il-8* and *myd88*

**Figure 6:**
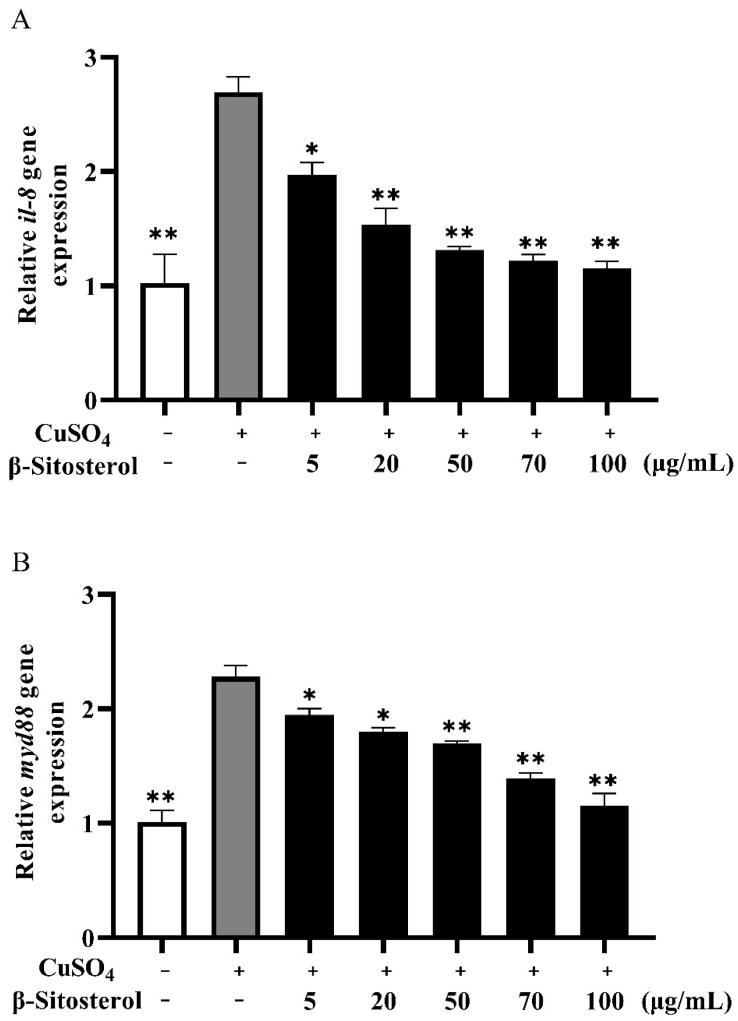
Relative expressions of immune response-related genes in zebrafish larvae after CuSO_4_ stimulation and β-sitosterol treatment: (**A**) relative expression of the *il-8* gene; (**B**) relative expression of the *myd88* gene. The data are expressed as the mean ± standard error based on three biological replicates. * *p* < 0.05 and ** *p* < 0.01 compared with the group treated with CuSO_4_ alone.

The expressions of the *il-8* gene and *myd88* gene in zebrafish stimulated by CuSO_4_ alone significantly increased compared with the blank control (Figure 6A,B). After 24 h of treatment with different doses of β-sitosterol, the expressions of *il-8* and *myd88* decreased. The expression of the *il-8* gene decreased significantly after treatment with 50, 70, and 100 μg/mL β-sitosterol, with significant decreases in the *myd88* expression in the 70 and 100 μg/mL β-sitosterol treatment groups.

## 4. Discussion

Cu^2+^ has been shown to cause oxidative stress [24,25]. Oxidative stress can occur through two mechanisms: (1) Cu^2+^ can directly damage DNA and cells and (2) Cu^2+^ can increase ROS release by activating phagocytic cells and increasing tissue damage [26]. Changes in ROS may induce the activation of activator protein-1 (AP-1) and nuclear factor-kappa B (NF-κB), both of which upregulate proinflammatory cytokines and chemokines that cause inflammation. Inflammation is the result of damages in zebrafish after Cu^2+^ stimulation. Oxidative stress and inflammation are related to the toxic mechanism of Cu^2+^. Therefore, we used a zebrafish CuSO_4_ inflammation model to study the antioxidant and anti-inflammatory effects of β-sitosterol. Our results show that CuSO_4_ stimulation in zebrafish resulted in an inflammatory response, while pretreatment with β-sitosterol reduced this inflammatory response and enhanced the anti-inflammatory and antioxidant capacity of zebrafish.

### 4.1. In Vivo Antioxidant Effect of β-Sitosterol

The antioxidant activity of β-sitosterol was verified in vitro by a DPPH assay, which is commonly used to evaluate the free radical scavenging activity of antioxidants due to the fact of its rapidity, reliability, and good repeatability [19,27]. The DPPH assay has been applied extensively to detect the antioxidant activity of pure antioxidant compounds and various plant extracts [28,29]. In this study, the free radical scavenging rate of 100 μg/mL β-sitosterol exceeded 40% (Figure 2), demonstrating the antioxidant effect of β-sitosterol. Although in vitro experiments are simple, fast, and inexpensive, they cannot completely replace in vivo animal testing [28]. CuSO_4_ can induce oxidative stress by inducing ROS [30]. Zebrafish are widely used as a model for studying anti-inflammatory research [20]. In this study, a zebrafish CuSO_4_ inflammatory model was used to confirm the antioxidant effect of β-sitosterol in vivo. Oxyflufen exposure has been shown to induce oxidative stress and inflammation in zebrafish by promoting ROS production, leading to acute kidney injury [31]. CuSO_4_ also stimulates ROS production and inhibits the expressions of antioxidant genes [32]. Therefore, we investigated the effects of β-sitosterol on ROS production and antioxidant gene expression. To exclude the effect of complexation between β-sitosterol and Cu^2+^, we first verified the complexation ability of β-sitosterol for Cu^2+^. The complexation rate between 100 μg/mL β-sitosterol and Cu^2+^ was only 7% (Figure 1), indicating that the complexation did not affect the subsequent experimental results. Subsequently, we demonstrated that CuSO_4_ immersion stimulated oxidative stress in zebrafish, while pretreatment with β-sitosterol produced an antioxidant effect. We then evaluated the ROS generation in zebrafish using H_2_DCFDA, a membrane-permeable fluorescent dye [26,33,34]. β-Sitosterol at concentrations of 50, 70, and 100 μg/mL significantly reduced the production of ROS in zebrafish (Figure 3B,C). Thus, β-sitosterol has a significant antioxidant effect and can reduce ROS production in zebrafish to protect against oxidative stress.

β-Sitosterol has been shown to demonstrate antioxidant activity without causing acute toxicity, subchronic toxicity, or abnormalities in the synthesis of antioxidant enzymes [35,36]. β-Sitosterol also increases the activities of superoxide dismutase (SOD) and glutathione peroxidase (GPX) while decreasing catalase activity [37]. Both SOD and GPX are antioxidant enzymes [38]. SOD is a primary substance that scavenges ROS [39]. GPX plays a crucial role against oxidative stress, which can turn toxic substances into innocuous products by scavenging their free radicals [40]. Thus, SOD and GPX can reflect the body’s ability to resist oxidative damage [41]. The extract of *Clerodendrum Cyrtophyllum* Turcz could increase the level of *sod* mRNA in zebrafish [20]. In addition, the extract of *Monascus purpureus* could increase the level of *sod* and *gpx* mRNA in LNCaP cells [42]. This research used the expression of the antioxidant genes *sod* and *gpx* to verify the antioxidant activity of the extract. Therefore, to further study the mechanism of the antioxidant effect, we measured the effects of β-sitosterol on the expressions of two zebrafish genes (superoxide dismutase *sod* and glutathione peroxidase *gpx4b*) involved in the antioxidant system. In this study, after 24 h of Cu^2+^ exposure, the expressions of *sod* and *gpx4b* were significantly lower compared with the control group. However, pretreatment with β-sitosterol significantly increased the expressions of the *sod* and *gpx4b* genes compared with group treated with CuSO_4_ alone. Pretreatment with 20, 50, 70, and 100 μg/mL β-sitosterol had the most obvious effects on *sod* expression (Figure 4A; *p* < 0.01), while pretreatment with 50, 70, and 100 μg/mL β-sitosterol had the most obvious effects on *gpx4b* expression (Figure 4B; *p* < 0.01). The effects of pretreatment with β-sitosterol at 70 and 100 μg/mL were particularly significant (Figure 4A,B). The effect of β-sitosterol on *sod* and *gpx4b* indicates that β-sitosterol has certain antioxidant effect in zebrafish after oxidative stress.

### 4.2. Anti-Inflammatory Effect of β-Sitosterol in Zebrafish

β-Sitosterol can inhibit NF-κB and extracellular signal-regulated kinases (ERK)/p38 mitogen-activated protein kinases (p38 MAPK) signal transduction to reduce *Escherichia coli* lipopolysaccharide-induced mouse microglial inflammatory response [43]. β-Sitosterol can also reduce the mRNA expressions of inflammatory factors, such as IL-6 and tumor necrosis factor-α (TNF-α) [43]. β-Sitosterol significantly reduced weight loss and colon length and alleviated the microscopic manifestations of dextran sulfate sodium-induced colitis in mice [15]. In J774A1 macrophages induced by lipopolysaccharide, β-sitosterol increased the activity of anti-inflammatory factor IL-10, decreased the activities of chemokines and pro-inflammatory factors, enhanced the activity of tyrosine phosphatase, and inhibited the migration of NF-κB [14]. In zebrafish larvae, exposure to CuSO_4_ can damage lateral neuromast hair cells and induce neutrophil migration to inflammatory foci. The temporary treatment of zebrafish larvae with CuSO_4_ selectively triggered the death of hair cells in the lateral line system and led to the rapid accumulation of neutrophils at the damaged site, resulting in a strong acute inflammatory response [44,45]. In this study, pretreatment with β-sitosterol before CuSO_4_ exposure significantly reduced the recruitment of neutrophils in the damaged parts of the zebrafish, and the number of neutrophils in the damaged parts gradually decreased with an increasing β-sitosterol concentration. These results confirm the good anti-inflammatory effect of β-sitosterol in zebrafish. While pretreatment with 5 μg/mL β-sitosterol did not significantly affect the number of neutrophils compared to the group treated with CuSO_4_ alone, the anti-inflammatory effects of the other concentrations of β-sitosterol, particularly 70 and 100 μg/mL (Figure 5B,C), were relatively significant. The gene *myd88* encodes a cytoplasmic adaptor protein that plays a critical role in innate and adaptive immune responses. This gene is also an essential signal transducer in the interleukin-1 and Toll-like receptor signaling pathways [46,47], which modulate the levels of pro-inflammatory mediators [48,49]. Abundant evidence suggests that certain pro-inflammatory cytokines, such as IL-1β, IL-2, TNF-α, IL-6, and IL-8, are involved in the pathological inflammatory process [50]. CXCL8 (IL-8) is an important chemokine that mediates neutrophil migration, aggregation, and function in inflammatory sites [51]. Meanwhile, *cxcl8-l1* (zebrafish *il-8a*) and *cxcl8-l2* (zebrafish *il-8b*) play major roles in neutrophil recruitment, which is a characteristic of acute inflammation [52]. Vitamin D significantly reduced *myd88* and *il-8* mRNA levels and inhibited lipopolysaccharide-induced inflammatory responses [53]. Therefore, in this study, we examined the expressions of *myd88* and *il-8* after zebrafish were treated with 10 μM (a nonlethal concentration) of CuSO_4_ for 24 h. The treatment with CuSO_4_ alone significantly increased the expressions of these genes compared with the blank control (Figure 6A,B). After β-sitosterol treatment for 24 h, the levels of *il-8* and *myd88* significantly decreased. Thus, β-sitosterol enhanced the innate immunity of the zebrafish and weakened the inflammatory response to CuSO_4_ stimulation. The inhibitory effects on the expressions of *myd88* and *il-8* were proportional to the concentration of β-sitosterol. However, whether the inhibition of neutrophil migration by β-sitosterol is really related to the inhibition of *il8* expression needs further experimental verification. In summary, the results demonstrate that β-sitosterol has a certain anti-inflammatory effect and can improve the ability of zebrafish to resist stress.

## 5. Conclusions

This study showed that β-sitosterol can reduce the production of ROS in a CuSO_4_-induced inflammation model in zebrafish and upregulate the expressions of *sod* and *gpx4b* to inhibit oxidative stress. Moreover, β-sitosterol can inhibit neutrophil migration to the lateral line and reduce the expressions of inflammatory genes (*il-8* and *myd88*) to inhibit inflammation. These results help explain the protective effect of β-sitosterol against CuSO_4_ toxicity in vivo. The findings support the application of β-sitosterol as a feed additive to improve the anti-inflammatory and antioxidant capacity of aquatic products.

## Figures and Tables

**Table 1:** Primers used in this study.

Gene Name	GenBank Accession No.	Forward and Reverse Primer Sequences (5′-3′)
*β-actin* [20]	AF057040	F: CCCCATTGAGCACGGTATTG R: ATACATGGCAGGGGTGTTGA
*il-8*	XM_009306855.3	F: GAGAGGTCTGGCTGTAGATC R: AGTTGTCATCAAGGTGGCAAT
*myd88*	NM_212814.2	F: GTGATGCCTGTGATTTTCAGACTAA R: CGGCCTCTTCATGGATTTGT
*sod* [20]	NM_131294.1	F: ATGGTGAACAAGGCCGTTTG R: AAAGCATGGACGTGGAAACC
*gpx4b* [20]	BC095133.1	F: TGAGAAGGGTTTACGCATCCTG R: TGTTGTTCCCCAGTGTTCCT

## Data Availability

Data is contained within the article.
